# Des(1–3)IGF-1 Treatment Normalizes Type 1 IGF Receptor and Phospho-Akt (Thr 308) Immunoreactivity in Predegenerative Retina of Diabetic Rats

**DOI:** 10.1080/15438600303729

**Published:** 2003

**Authors:** A. Kummer, B. E. Pulford, D. N. Ishii, G. M. Seigel

**Affiliations:** 1 University of Rochester School of Medicine and Dentistry Rochester New York USA; 2 Colorado State University Fort Collins Colorado USA; 3 Department of Ophthalmology, Physiology and Biophysics University at Buffalo the State University of New York 3435 Main Street, Sherman 124 Buffalo NY 14214 USA

## Abstract

Little is known about interventions that may prevent predegenerative changes in the diabetic retina. This study tested the hypothesis that immediate, systemic treatment with an insulin-like growth factor (IGF)-1 analog can prevent abnormal accumulations of type 1 IGF receptor, and phospho-Akt (Thr 308) immunoreactivity in predegenerative retinas of streptozotocin (STZ) diabetic rats. Type 1 IGF receptor immunoreactivity increased approximately 3-fold in both inner nuclear layer (INL) and ganglion cell layer (GCL) in retinas from STZ rats versus nondiabetic controls. Phospho-Akt (Thr 308) immunoreactivity increased 5-fold in GCL and 8-fold in INL of STZ rat retinas. In all cases, immunoreactive cells were significantly reduced in STZ des(1–3)IGF-1–treated versus STZ rats. Preliminary results suggested that vascular endothelial growth factor (VEGF) levels may also be reduced. Hyperglycemia/ failure of weight gain in diabetic rats continued despite systemic des(1–3)IGF-1. These data show that an IGF-1 analog can prevent early retinal biochemical abnormalities implicated in the progression of diabetic retinopathy, despite ongoing hyperglycemia.


*Experimental Diab. Res.*, 4:45–57, 2003
Copyright © 2003 Taylor & Francis
1543-8600/03 $12.00 + .00
DOI: 10.1080/15438600390214626

Taylor&Francis healthsciences

### Des(1–3)IGF-1 Treatment Normalizes Type 1 IGF Receptor and Phospho-Akt (Thr 308) Immunoreactivity in Predegenerative Retina of Diabetic Rats

**A. Kummer,[1] B. E. Pulford,[2] D. N. Ishii,[2] and G. M. Seigel[1]**

[1]*University of Rochester School of Medicine and Dentistry, Rochester, New York, USA*
[2]*Colorado State University, Fort Collins, Colorado, USA*

**Little is known about interventions that may prevent predegenerative changes in the diabetic retina. This study tested the hypothesis that immediate, systemic treatment with an insulin-like growth factor (IGF)-1 analog can prevent abnormal accumulations of type 1 IGF receptor, and phospho-Akt (Thr 308) immunoreactivity in predegenerative retinas of streptozotocin (STZ) diabetic rats. Type 1 IGF receptor immunoreactivity increased approximately 3-fold in both inner nuclear layer (INL) and ganglion cell layer (GCL) in retinas from STZ rats versus nondiabetic controls. Phospho-Akt (Thr 308) immunoreactivity increased 5-fold in GCL and 8-fold in INL of STZ rat retinas. In all cases, immunoreactive cells were significantly reduced in STZ des(1–3)IGF-1–treated versus STZ rats. Preliminary results suggested that vascular endothelial growth factor (VEGF) levels may also be reduced. Hyperglycemia/failure of weight gain in diabetic rats continued despite systemic des(1–3)IGF-1. These data show that an IGF-1 analog can prevent early retinal biochemical abnormalities implicated in the progression of diabetic retinopathy, despite ongoing hyperglycemia.**

Received 4 October 2002; accepted 2 February 2003.

The present address of A. Kummer is Department of Ophthalmology, University of Edinburgh, The Princess Alexandra Eye Pavilion, Chalmers Street, Edinburgh, EH3 9HA, UK.

This work was supported in part by the Diabetes Action Research and Education Foundation (GMS) and NIDDKD grant R01 DK53922 (DNI).

The authors thank Lorrie Campbell for technical support and Janet Wagner for assisting with histological specimens.

Address correspondence to Gail M. Seigel, PhD, Department of Ophthalmology, Physiology and Biophysics, University at Buffalo, the State University of New York, 3435 Main Street, Sherman 124, Buffalo, NY 14214, USA. E-mail: gseigel@frontiernet.net

**Keywords** Apoptosis; Blood-Retinal Barrier; Diabetic Retinopathy; Experimental Diabetes; Growth Factors; Immunohistochemistry; Immunopathology; Retina; Retinal Degeneration; Streptozotocin

The pathogenesis of diabetic retinopathy is a complex process involving ischemic/hyperglycemic and growth factor retinal insults that can result in neovascularization and vision loss. The incidence of diabetic retinopathy can be reduced somewhat when blood glucose is well-controlled [1]. Although early glucose control may be important in delaying the onset of diabetic retinopathy, glucose control alone, unfortunately, cannot halt the eventual progression of retinopathy [2]. There is a pressing need for novel interventions to supplement glycemic control.

Insulin-like growth factor-1 (IGF-1) is among several factors that have been suggested to regulate predegenerative abnormalities, including early elevation of vascular endothelial growth factor (VEGF) levels in the retina [3, 4]. VEGF has been identified as a causative factor in retinal neovascularization as well as vascular permeability [5, 6] associated with proliferative diabetic retinopathy. There is controversy as to whether serum or vitreous IGF-1 levels correlate with the progression of retinal neovascularization in clinical diabetes. Some studies report no correlation [7], whereas others report correlations with either elevated or decreased levels of IGF-1 in the vitreous or serum of diabetic patients with retinopathy [8–10]. Disparities in these reports may be due to the methods and biological samples used for analysis (mRNA versus protein, vitreous versus serum), but also to differences in the extent of blood-retinal barrier (BRB) breakdown at the time of sample collection. Recent studies point to increased permeability of serum IGF-1 in proliferative retinopathy as the main source of vitreous IGF-1. In the only study conducted to date in which both VEGF and IGF-1 were measured in the vitreous of patients with proliferative diabetic retinopathy, VEGF levels were elevated, whereas free IGF-1 levels were reduced when corrected for protein infiltration [9]. This study suggests that IGF-1 is not correlated with proliferative retinopathy and its role in retina needs to be clarified.

Circulating IGF-1 levels are reduced in diabetic patients [11] and rodents. There are early alterations in visual function in the absence of retinopathy in diabetic patients [10], and retinal neuron loss in clinical and experimental diabetes [13, 14]. Recent studies show that early administration of replacement doses of IGF-1 can prevent certain diabetic complications, such as neuropathy in diabetic rats [15–17]. IGF-1 or its analogues can inhibit neuroretinal cell death caused by hypoxia in culture [18], and IGF-1 supports neurite outgrowth and survival in amacrine neurons [19]. Moreover, administration of low replacement doses of IGF-1 (20 to 40 $\mu$g/kg/day) for 24 weeks did not cause progression of retinopathy in a phase II trial of 53 type 1 diabetic patients [20]. These data show that IGF-1 administration is relatively safe, and early IGF-1 treatment might prevent diabetic complications in the eyes as well as nerves. It is not known whether IGF-1 sequestration to IGF-binding proteins (IGFBPs) is necessary for effective treatment. Des(1–3)IGF-1 is an IGF-1 analogue lacking the N-terminal tripeptide, which has greatly reduced affinity for IGFBPs.

Additional studies are needed to determine the early biochemical pathology in the diabetic eye. To this end, acute biochemical changes were investigated in the streptozotocin (STZ) rat. The purpose of this study was to test the hypotheses that administration of des(1–3)IGF-1 at the time of onset of diabetes can (i) normalize the type 1 IGF receptor levels in retina, (ii) inhibit the phospho-Akt (Thr 308) retinal stress response, and (iii) prevent these predegenerative biochemical abnormalities independently of poor glycemic control. In order to test whether des(1–3)IGF-1 could prevent the onset of acute biochemical abnormalities, treatment with this IGF-1 analogue was initiated at the time of induction of diabetes. By using des(1–3)IGF-1, this study additionally tests the hypothesis that IGF-1 sequestration to IGFBP is not essential for preventing at least certain diabetic complications.

### METHODS

### Materials

STZ and Glucose Diagnostic Kit 510A were purchased from Sigma Chemical (St. Louis, MO). Des(1–3)IGF-1 was from GroPep (Adelaide, Australia). Primary rabbit polyclonal antibody against Phospho-Akt (Thr 308) (Cell Signaling Technologies, Beverly, MA) as well as mouse monoclonal antibodies against type 1 IGF receptor (Calbiochem, San Diego, CA) and VEGF (Calbiochem) were obtained. Alzet osmotic minipumps (0.5 $\mu$L/h; 2-week duration) were from Durect (Cupertino, CA).

### Animals

Animal experiments were performed in accordance with National Institutes of Health (NIH) guidelines (DHEW publication NIH80-23). Sprague-Dawley (Harlan Sprague-Dawley, Indianapolis, IN) male rats were maintained on 20 g per day of rat chow until the study, and chow and water were provided ad libitum thereafter. Rats (12 weeks old) were randomly assigned to treatment groups (5 rats per group). All solutions to be administered to rats were sterilized by passage through 0.2-$\mu$m Acrodisk filters (Pall Corp., Ann Arbor, MI). Diabetes was induced by intraperitoneal [IP] administration of 50 mg/kg STZ, whereas nondiabetic rats were administered solvent (10 mM sodium citrate in 0.9% NaCl, pH 4.5). The treatment groups were as follows: ND, non-diabetic; STZ-veh, diabetic with subcutaneous osmotic minipumps releasing vehicle (1 mM acetate, pH 6) for 2 weeks; or STZ-des, diabetic rats with pumps releasing des(1–3)IGF-1 (5 $\mu$g/rat/day) for 2 weeks. Two weeks later, the animals were euthanized, and the eyes were placed in 4% paraformaldehyde in phosphate-buffered saline (PBS). The fixed eyes were embedded in paraffin and cut into 4-$\mu$m-thin sections. Tail blood was withdrawn for glucose assays 1 day after STZ or vehicle treatment as well as at 2 weeks.

### Immunohistochemistry

Paraffin-embedded retinal tissue sections were rehydrated through xylene and a series of graded alcohol concentrations. Tissue sections were incubated in 0.25% Triton X-100 for 5 minutes. After a rinse in PBS, sections were incubated for 1 hour with primary antibody. After rinsing 3 $\times$ 5 minutes in PBS, sections were incubated with a 1:1500 dilution of biotinylated goat anti-rabbit or anti-mouse immunoglobulin (Vector Laboratories, Burlingame, CA) for 60 minutes. Tissue sections were incubated for 20 minutes with horseradish peroxidase–conjugated avidin (Elite kit, Vector Laboratories). The sections were rinsed in 0.05 M Tris and antigens were detected with a diaminobenzidine (DAB) kit (Pierce); the brown/black reaction product was visualized by light microscopy. Negative controls consisted of incubation with 5% goat serum without primary antibody, and did not generate any detectable reaction product. After staining, immunoreactive cells in the ganglion cell layer (GCL) and the inner nuclear layer (INL) were counted in 3 random 500-$\mu$m-long segments of the 4-$\mu$m-thick retinal cross-sections taken from each eye of the rat. There were approximately 100 cells along the length of each 500-$\mu$m segment. Sample labels were not visible to observers at the time of cell counting.

### Statistical Analysis

Results are expressed as means ± SD for numbers of immunoreactive cells per 500-$\mu$m segment for each treatment group. Cell counts were analyzed with Fisher's post hoc least significant differences test. Differences between group means were accepted as significant at $P < .05$.

### RESULTS

A 2-week duration of STZ diabetes was selected for this study to examine early pathological changes that may precede degenerative events. An immunohistological approach was chosen in order to identify the specific retinal cell layers affected.

### Des(1–3)IGF-1 Treatment Did Not Prevent Hyperglycemia Nor Weight Loss in Diabetic Rats

Excessively high levels of IGF-1 or IGF analogues may cross-occupy the insulin receptor and ameliorate weight loss and hyperglycemia. The low dose of des(1–3)IGF-1 used in this experiment was not expected to alter these parameters; nevertheless, measurements were taken and the results are shown in Figure 1.

As seen in Figure 1 (*top panel*), significant differences in weight loss were not observed in des(1–3)IGF-1 versus vehicle-treated diabetic groups. Nondiabetic rats gained approximately 51 g, whereas diabetic rats weighed significantly less. No difference in weight was observed between STZ-veh and STZ-des groups. At termination of the experiment, serum glucose concentrations were measured as well (Figure 1, *bottom panel*). The diabetic rats were clearly hyperglycemic. Des(1–3)IGF-1 treatment did not reduce hyperglycemia in diabetic rats.

### Effect of Des(1–3)IGF-1 Treatment on Type 1 IGF Receptor Immunoreactivity

There was a low level of IGF-1 receptor immunoreactivity in the GCL, INL, and BRB in the nondiabetic retina (Figure 2*A*). Immunoreactivity in all of these areas appeared to be increased in retinas from STZ-veh rats (Figure 2*B*). On the other hand, such immunoreactivity appeared to be reduced in STZ-des versus STZ-veh retinas, and was similar to that of the nondiabetic group (Figure 2*C*).

To determine whether these differences were significant, type 1 IGF receptor–immunoreactive cells were counted in the GCL and INL in retinas from all rats. Type 1 IGF receptor immunoreactivity was significantly increased ($P < .0001$) in both the GCL (Figure 3*A*) and INL (Figure 3*B*) in STZ-veh versus the nondiabetic group. With des(1–3)IGF-1 treatment, type 1 IGF receptor immunoreactivity returned nearly to control levels. Immunoreactivity was significantly reduced ($P < .0001$) in STZ-des versus STZ-veh groups (Figure 3*A*, *B*).

### Preliminary Studies on Effect of Des(1–3)IGF-1 Treatment on VEGF Immunoreactivity

In anticipation of future studies, an initial examination of VEGF immunoreactivity was performed to determine whether des(1–3)IGF-1 treatment might prevent an increase in VEGF immunoreactivity. Adjacent sections of retinal tissue from the foregoing experiments were examined. The nondiabetic control group showed a basal level of VEGF immunoreactivity that was mainly associated with retinal endothelial cells (Figure 4*A*). A qualitative change was observed in STZ-veh rats, and VEGF immunoreactivity appeared in retinal pigmented epithelial cells (RPEs) (Figure 4*B*). This increase in RPE-associated VEGF immunoreactivity was prevented by treatment of diabetic rats with des(1–3)IGF-1 (Figure 4*C*). Occasional cells of the inner retina, however, stained positively for VEGF in STZ-des as well as ND control tissues.

### Effect of Des(1–3)IGF-1 Treatment on Phospho-Akt (Thr 308) Immunoreactivity

There was a basal level of the apoptotic-stress response protein phospho-Akt (Thr 308) immunoreactivity in the GCL and INL in the nondiabetic retina (Figure 5*A*). Immunoreactivity in both of these areas appeared to be increased in the retina from STZ-veh rats (Figure 5*B*). On the other hand, such immunoreactivity appeared to be reduced in STZ-des versus STZ-veh retinas, and was similar to the ND group (Figure 5*C*).

To determine whether these differences were significant, phospho-Akt (Thr 308) immunoreactive cells were counted in the GCL and INL in retinas from all rats. Immunoreactivity was significantly increased ($P < .0001$) in both the GCL (Figure 6*A*) and INL (Figure 6*B*) in STZ-veh versus nondiabetic groups. With des(1–3)IGF-1 treatment, phospho-Akt (Thr 308) immunoreactivity returned nearly to control levels. Immunoreactivity was significantly reduced ($P < .0001$) in STZ-des versus STZ-veh groups (Figure 6*A*, *B*).

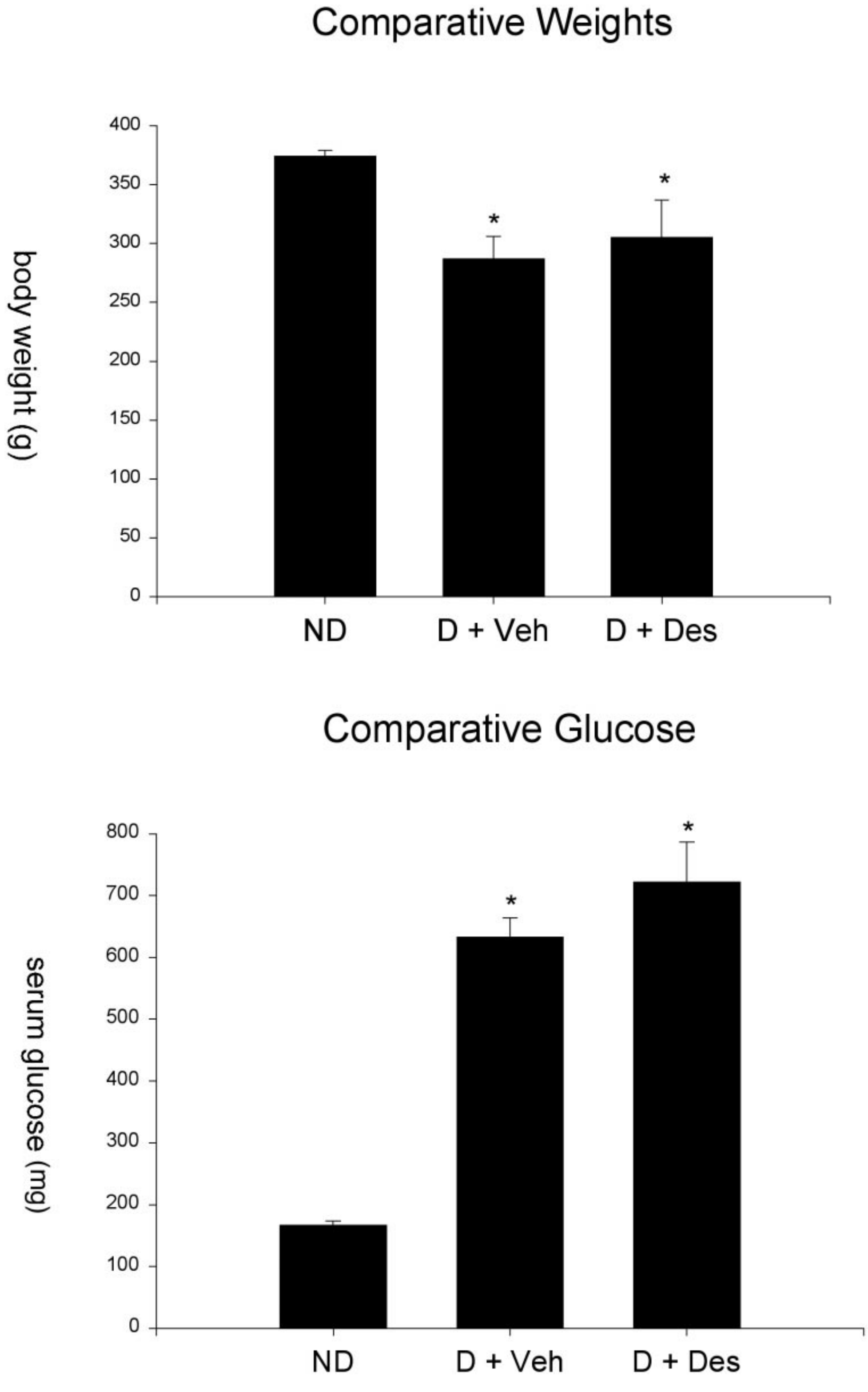

**FIGURE 1**

Effect of des(1–3)IGF-1 administration on body weights and serum glucose levels of diabetic rats. Streptozotocin diabetic rats (12 weeks old) were implanted with subcutaneous pumps that released either vehicle (D + Veh) or 5 $\mu$g/day des(1–3)IGF-1 (D + Des) for 2 weeks. Untreated nondiabetic rats were also studied (ND). *Top panel*, body weights; *bottom panel*, serum glucose content. ND, 9.1 ± 0.7 mmol/L; D + Veh, 32.0 ± 1.6 mmol/L; D + Des, 38.5 ± 3.7 mmol/L. Values are means ± SEM. $^*P < .05$ for ND versus (D + Veh) or D + Des groups.

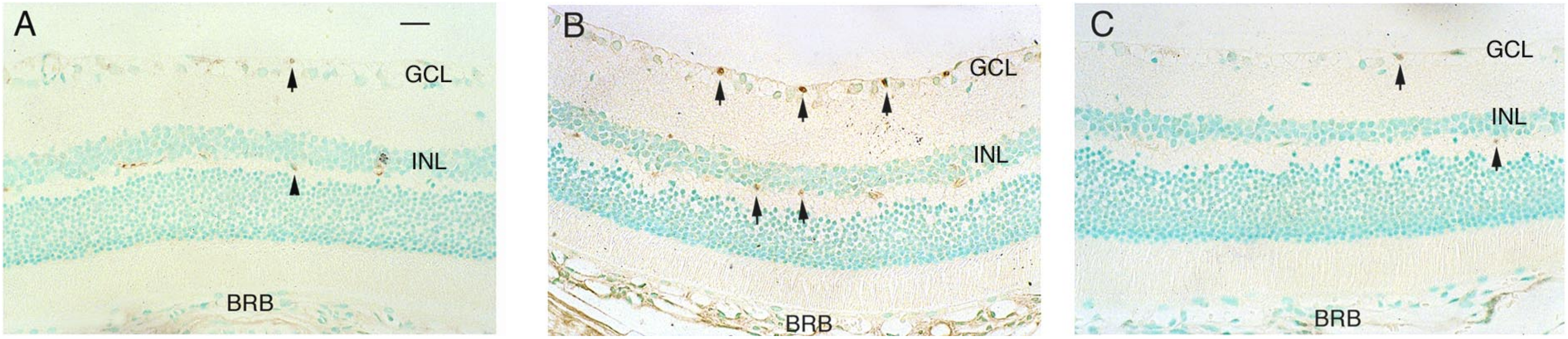

**FIGURE 2**

Profile of type 1 IGF receptor immunoreactivity in retina from diabetic rats treated with or without des(1–3)IGF-1. Alternate sections of retinal tissue from nondiabetic control, STZ-veh, and STZ-des rats were stained with type 1 IGF receptor antibody (1:100 dilution). (*A*) Control tissue demonstrates few IGF-1–immunoreactive cells in the GCL and INL (*arrowheads*). (*B*) STZ-veh rat retinal tissue reveals several highly immunopositive retinal ganglion cells (*arrowheads*), as well as immunoreactivity at the BRB. (*C*) STZ-des rat retina with few immunopositive cells (*arrowheads*), as well as decreased immunoreactivity at the BRB. Immunostaining was absent when primary antibody was omitted and replaced with 5% normal goat serum. Magnification bar = 50 $\mu$m.

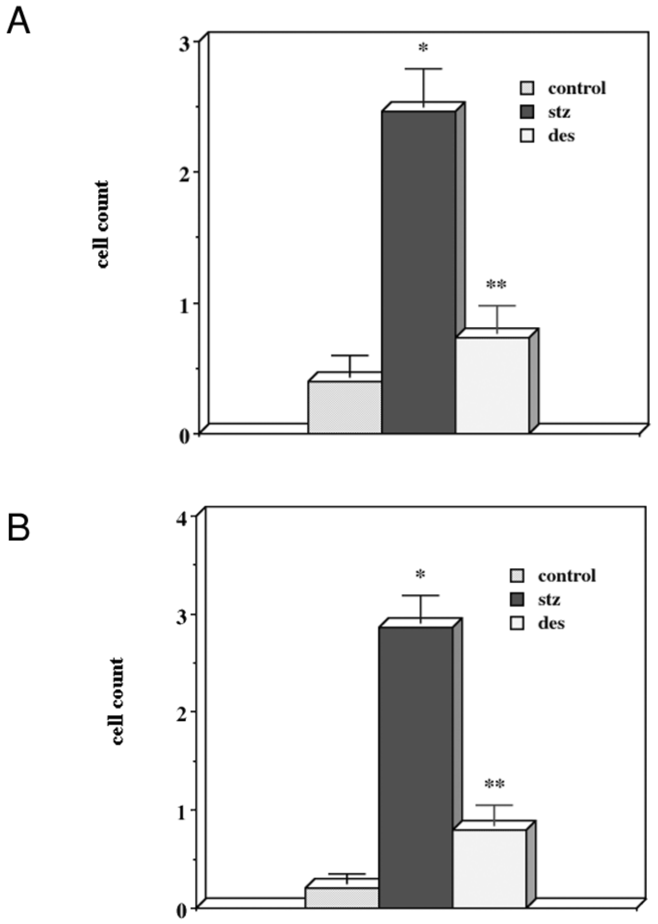

**FIGURE 3**
Type 1 IGF receptor immunoreactivity was increased in STZ rat retina, and such increase was prevented by des(1–3)IGF-1 treatment. The mean numbers of type 1 IGF receptor immunoreactive cells per 500-$\mu$m-length retinal sections were calculated: (*A*) Ganglion cell layer (GCL) ($^{*}P < .0001$ STZ vs. control, $^{**}P < .0001$ des vs. control) and (*B*) inner nuclear layer (INL) ($^{*}P < .0007$ STZ vs. control, $^{**}P < .0027$ des vs. STZ). The mean immunoreactive cell count was significantly increased in STZ-veh versus nondiabetic control retina ($P < .0001$). This count was significantly reduced in STZ-des versus STZ-veh retina ($P < .0001$). Error bars indicate $\pm$ SD.

### DISCUSSION

Type 1 IGF receptor and phospho-Akt (Thr 308) immunoreactivity were increased in the GCL and INL of the rat retina 2 weeks after induction of diabetes with STZ. These changes, seen at 2 weeks, are among the earliest biochemical abnormalities that have been detected in the eye in diabetes, which coincide with VEGF up-regulation and BRB breakdown [3, 4]. These predegenerative biochemical abnormalities were prevented by the subcutaneous administration of des(1–3)IGF-1 at time of onset of diabetes. Interestingly, treatment with des(1–3)IGF-1 was effective independently of poor metabolic control. These data suggest that treatment with IGF-1 or its analogues may be protective if administered early in the course of diabetes. There is evidence for synergistic effects between IGF-1 and VEGF on retinal endothelial cell proliferation/survival [21], and concern remains regarding the potential neovascularizing effects of IGF-1 in the retina. The present acute study brings new data suggesting that the role of IGF may be more complex than previouslyappreciated.

### The Pathophysiology of Diabetic Neurological Disturbances is Mimicked by a Reduction of IGF Activity in Nondiabetic Conditions and IGF-1 Administration May Protect Against Deleterious Effects of IGF Depletion in Diabetes

Reduced axonal diameters, diminished conduction velocity, impaired nerve regeneration, and neuronal death are major pathological features of clinical diabetic neurological disturbances. A reduction of IGF activity in *nondiabetic* animals can mimic these effects. For example, anti-IGF antibodies impair nerve regeneration [22, 23], and administration of anti-IGF antibodies or IGFBPs can cause neuronal death [24]. IGF-1–null mice have reduced axon diameters and nerve conduction velocity [25] as well as neuron loss [26]. IGF-1 and IGF-2 mRNA levels are reduced in various tissues, including nerves, brain, and spinal cord in diabetic rodents [16, 27, 28]. IGF-1 gene expression is reduced in retina from diabetic patients and rodents [29]. IGF-1 gene expression is reduced in liver, the primary source of circulating IGF-1 [30, 31], and circulating IGF-1 levels are reduced in diabetic rats [32, 33], as well as in type 1 and type 2 diabetic patients [34, 35]. Thus, there is a profound loss of IGF-1 support for various tissues early in diabetes. In the present study, immediate des(1–3)IGF-I treatment protected against early predegenerative changes in retina.

### Des(1–3)IGF-1 Treatment is Effective Despite Poor Metabolic Control

The earliest detection of retinal neural degeneration in STZ diabetic rats is 4 weeks [13]. Consequently we examined for predegenerative changes at 2 weeks after the induction of diabetes. Des(1–3)IGF-1 protected against predegenerative retinal abnormalities independently of poor metabolic control evidenced by continued hyperglycemia and failure of weight gain. This suggests that the early predegenerative biochemical changes that were observed were possibly not a consequence of acute hyperglycemia per se. Alternatively, these

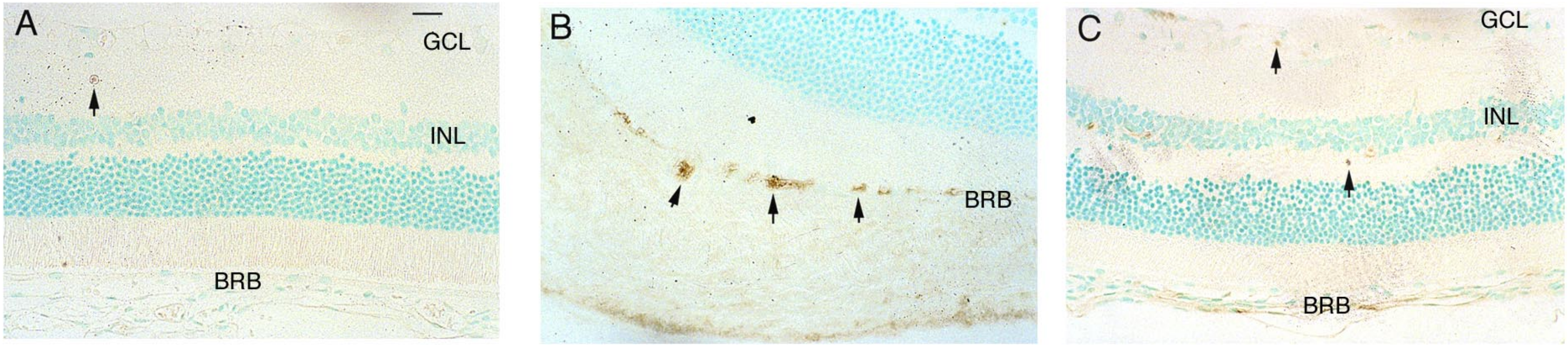

**FIGURE 4**

Representative profiles of VEGF immunoreactivity in the retinal pigmented epithelial cells of diabetic rats treated with or without des(1–3)IGF-1. Histological sections of retinal tissue from nondiabetic control, STZ-veh, and STZ-des rats were stained with VEGF antibody (1:100 dilution) as described in Methods. (*A*) Nondiabetic retinal section with small blood vessel positively stained for VEGF (*arrowhead*). (*B*) STZ-veh section shows emergence of several VEGF-immunopositive retinal pigmented epithelial (RPE) cells (*arrowheads*). (*C*) STZ-des rat retina with no evidence of VEGF-immunoreactive RPE cells. As with *A*, some small blood vessels stain positively for VEGF (*arrowheads*). These data are representative of 15 retinal sections, with immunostaining repeated three times. When VEGF antibody was omitted and replaced with 5% normal goat serum, immunostaining was absent (not shown). Magnification bar = 50 $\mu$m.

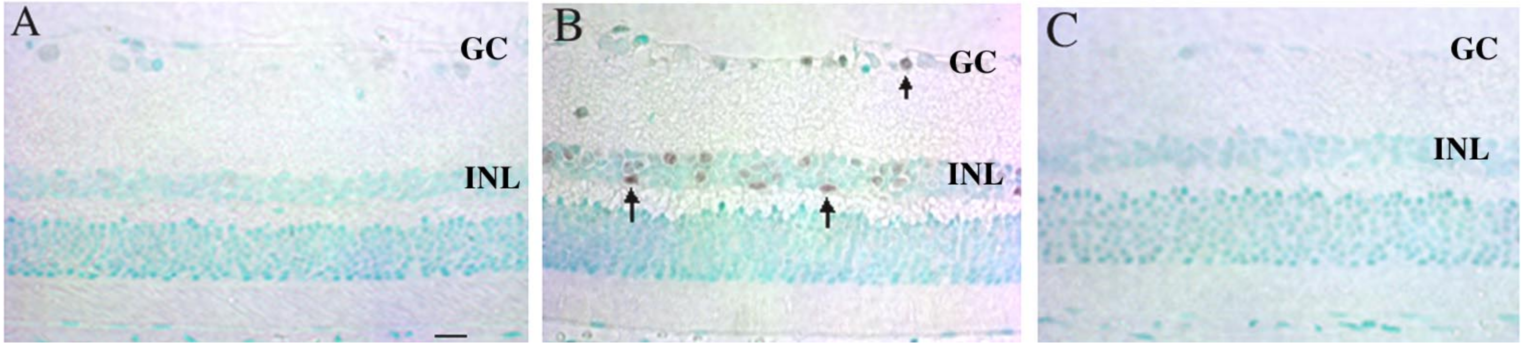

**FIGURE 5**

Profile of phospho-Akt (Thr 308) immunoreactivity in retina from diabetic rats treated with or without des(1–3)IGF-1. Alternate sections of retinal tissue from nondiabetic control, STZ-veh, and STZ-des rats were stained with phospho-Akt (Thr 308) antibody (1:100 dilution). (*A*) Control tissue demonstrates little to no phospho-Akt immunoreactive cells in the GCL and INL. (*B*) STZ-veh rat retinal tissue reveals several highly immunopositive retinal ganglion cells (*arrowheads*). (*C*) STZ-des rat retina with no immunopositive cells in the field. Immunostaining was absent when primary antibody was omitted and replaced with 5% normal goat serum. Magnification bar = 50 $\mu$m.

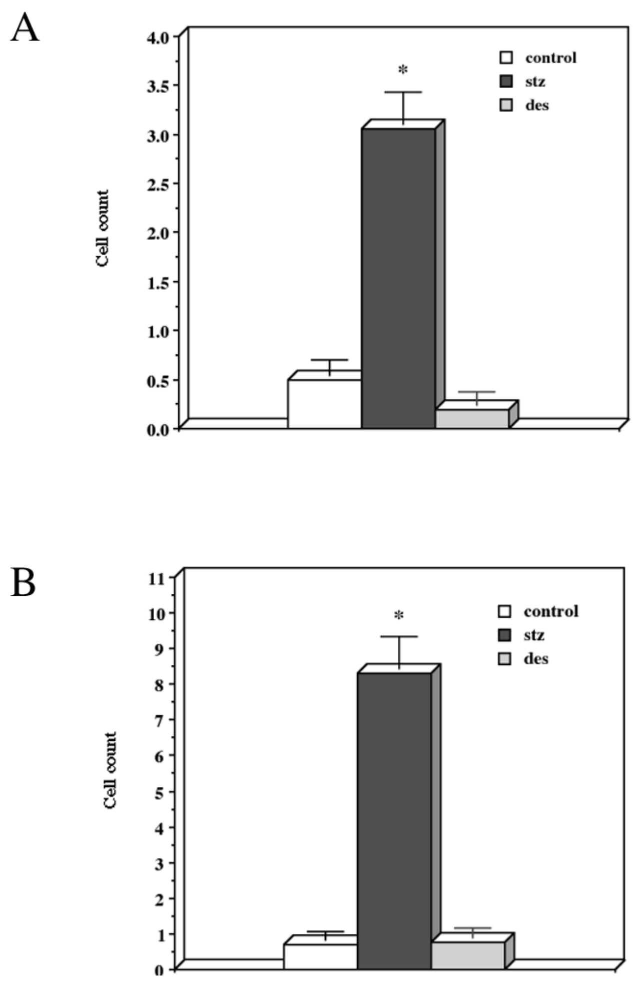

**FIGURE 6**
Phospho-Akt (Thr 308) immunoreactivity was increased in STZ rat retina, and such increase was prevented by des(1–3)IGF-1 treatment. The mean numbers of type 1 IGF receptor immunoreactive cells per 500-$\mu$m-length retinal sections were calculated: (*A*) Ganglion cell layer (GCL) and (*B*) inner nuclear layer (INL). The mean immunoreactive cell count was significantly increased in STZ-veh versus nondiabetic control retina ($P < .0001$). This count was significantly reduced in STZ-des versus STZ-veh retina ($P < .0001$). Error bars indicate $\pm$ SD.

abnormalities were a consequence of the loss of IGF-1 activity in diabetes. This is consistent with the observation that low doses of IGFs prevent diabetic neuropathy in type 1 [9, 15] diabetic rats despite hyperglycemia and weight loss, and in type 2 [16] diabetic rats despite hyperglycemia and weight gain. The present studies show that des(1–3)IGF-1 treatment was effective independently of continued weight loss.

It might be considered that large pharmacologic doses of IGF-1 can cross-occupy the insulin receptor and reduce hyperglycemia. This occurs at doses that exceed by severalfold the 31-$\mu$g/rat/day IGF-1 production in liver. By contrast, the des(1–3)IGF-1 dose used in this study (5 $\mu$g/rat/day), and the 4.8 $\mu$g/rat/day IGF-1 used elsewhere [9, 15], were too low to ameliorate hyperglycemia in diabetic rats.

### IGFBP May Not be Essential for Protection

Des(1–3)IGF-1 is a naturally occurring truncated form of IGF-1 that is missing the N-terminal tripeptide important for binding to IGFBP [36, 37]. Consequently, it is more potent than IGF-1 in vitro [38] and in vivo [33] due to reduced sequestration to IGFBP. It binds with 25-fold lower affinity to IGFBP-3 [39], has markedly reduced affinity for IGFBP-1, and 40-fold lower affinity to IGFBP-4 and -5 but retains similar affinity for the type 1 IGF receptor [39–42]. The data in the present study suggest that IGF-1 binding to IGFBP is not essential for protection against early predegenerative changes in type 1 IGF receptor and phospho-Akt (Thr 308) levels in retina.

### IGF-1 Presence in Diabetic Retina

Elevated IGF-1 levels in the retina do not seem to originate from the retina itself, because IGF-1 mRNA levels are actually reduced in retinas from patients with 7-year duration diabetes as well as rats with 3- to 7-week duration STZ diabetes [29, 43]. The predominant source of the elevated vitreous IGF-1 levels is a breakdown of the BRB because various serum proteins are increased in the vitreous together with IGF-1, although at least some of the IGF-1 may be of intraocular origin [10, 44, 45].

Circulating IGF-I levels are reduced 50% in type 1 and type 2 diabetic patients [34]. Despite this decrease, serum IGF-1 levels remain at least 20- to 50-fold higher than vitreous IGF-1 levels; hence, the increased permeability of retinal capillaries in diabetes may contribute to the elevated total IGF-1 levels in the eye. Therefore, vitreous IGF-1 levels may initially be reduced in early stages of diabetes as a consequence of reduced retinal IGF-1 mRNA levels in patients and rats. With chronic diabetes, increased permeability may result in elevated vitreous IGF-1 levels. Factors that influence the rate at which the BRB breaks down may explain at least in part the variability in vitreous IGF-1 levels reported in various clinical studies [7–10].

### Type 1 IGF Receptor in Diabetes

The IGF-1 receptor (IGF-1R) appears to be under complex regulation in diabetes. In diabetes, IGF-1R mRNA levels are reduced in rat superior cervical ganglia [46], heart [47], and muscle [48], whereas IGF-1R protein levels are decreased in rat hippocampus [49]. Yet, retina [50] and endothelial cells cultured from human retina [50] have elevated levels of IGF-1R immunoreactivity. Consistent with this observation, the present results show that IGF-1R immunoreactivity was significantly increased in vivo in retina from STZ-veh versus nondiabetic rats (Figures 4, 5), and the diabetic rat may provide a model for studying this biochemical abnormality. Immediate des(1–3)IGF-1 administration prevented this increase in IGF-1R immunoreactivity in diabetic rats (Figures 4, 5); but it is unclear whether this effect is at the transcriptional or translational level. Increased receptor immunoreactivity in the early stages of experimental diabetes does not appear to result from hyperglycemia because it is prevented by des(1–3)IGF-1 irrespective of ongoing hyperglycemia.

These results are discordant with the observation that IGF-1R immunoreactivity is not increased in retina from 8-week diabetic rats [43]. This difference is possibly due to 8- versus 2-week duration of STZ diabetes. Permeability of the BRB is increased after 3-week STZ diabetes; perhaps the associated increase in vitreous IGF-1 levels [45] may lead to down-regulation of IGF-1R immunoreactivity in chronic disease.

### Putative Effect of Des(1–3)IGF-1 Treatment on VEGF

VEGF is among the leading candidates as the primary mediator of proliferative retinopathy. It can induce vascular endothelial cell proliferation, migration, and vasopermeability. Inhibitors of phosphorylation mediated by the VEGF receptor can completely block retinal neovascularization [51].

VEGF may accumulate in the retina from retinal and vascular sources. VEGF accumulates in the vitreous humor of patients with proliferative diabetic retinopathy [52]. Patients with proliferative diabetic retinopathy have increased VEGF mRNA content in the GCL, INL, and outer nuclear layer, and this seems to be associated with ischemic regions of retina [53]. VEGF immunoreactivity may occur early, prior to evidence of retinal ischemia [54]. An increase in VEGF mRNA is also observed in the GCL and INL in the retina of STZ diabetic rats [32, 55]. The increase in immunoreactive VEGF labeling is associated with increasing breakdown of the BRB, and is most prominent in the nerve fiber layer near the optic disk and in perivascular areas in diabetic rats [56]. These are the sites of BRB breakdown and neovascularization observed clinically. Early up-regulation of VEGF in diabetic retina is also associated with antioxidative defense mechanisms [57] and the formation of advanced glycation end products [58]. Hypoxia/ischemia, characteristic of diabetic retinal tissues, is a strong inducer of VEGF and may contribute to the activation of oxidative stress mechanisms in the diabetic retina (for review, see [59]). Our own previous studies have shown that neuroretinal cell death under hypoxic conditions can be inhibited by IGF-1 and its analogues in vitro [60].

Our VEGF immunostaining (Figure 4) showed a clustered pattern, which unfortunately did not lend itself to quantitative counts of random retinal fields. Yet, treatment with des(1–3)IGF-1 appeared consistently to reduce RPE-associated VEGF immunoreactivity. Consequently, these morphological data should be viewed with caution. Our preliminary results seem to indicate that the increase in VEGF immunoreactivity in the perivascular regions of retinas of diabetic rats is reduced by the administration of des(1–3)IGF-1 (Figure 4). This is potentially due to reduced VEGF permeability, or other causes. Further studies are underway.

### Phospho-Akt (Thr 308) and the Diabetic Stress Response

The serine/threonine protein kinase Akt (also known as PKB and Rac) plays a critical role in regulating the balance between survival and apoptosis in a variety of systems [61, 62]. In the context of the present study, it is also noteworthy that Akt is proposed to be an important downstream target of phosphoinositol (PI) 3-kinase in insulin-mediated processes. There are high levels of PKB-$\beta$ expression in insulin-responsive adipose tissue [63], whereas PKB-$\beta$–deficient mice exhibit manifestations of type II diabetes, including hyperglycemia and insulin resistance [64]. Mechanical stretch of retinal pericytes, proposed to exacerbate diabetic retinopathy, is also associated with increases in expression of both VEGF and activated phospho-Akt [65].

Akt phosphorylation was somewhat enhanced (123%) in STZ diabetic and galactosemic rats versus control, as measured by Western immunoblot of whole retinal cell lysates [43]. In our study, the differences are much more striking between control and diabetic retinal tissues, due to the specificity of our analysis of GCL and INL regions of the retina. Our results support the Gerhardinger suggestion that increased activation/phosphorylation of Akt may reflect a stress response in the retina, possibly through a p38/HOG1 kinase cascade of events [32, 50, 66]. Des(1–3)IGF-1 can cross the blood–central nervous system barrier [67], and might also cross the BRB. Consequently, one attractive interpretation of these data is that des(1–3)IGF-1 may have entered the eye and affected the phosphorylation and activation state of Akt. In our own previous studies [68], we have shown that the ability of insulin to rescue retinal cell cultures from cell death is mediated through the PI 3-kinase/Akt pathway, by the inhibition of caspase-3 activation. Therefore, the present observation that increased phospho-Akt (Thr 308) immunoreactivity is an early event in the course of STZ-induced diabetes appears to be important to our understanding of cell signaling and cell death mechanisms in diabetes-associated retinal degeneration. In fact, preliminary data from a separate, longer-term experiment show that apoptotic cell death is elevated and IGF-1 administration prevents such elevation in retina from diabetic rats (Seigel et al., unpublished data). This implies strongly that preventing these early predegenerative changes in diabetic retina may prevent the loss of retinal cells. The identification of predegenerative diabetic changes offer potential targets for future interventions, including therapy possibly with IGF-1 and its analogs.

### REFERENCES

[1] Vijan, S., Hofer, T. P., and Hayward, R. A. (1997) Estimated benefits of glycemic control in microvascular complications in type 2 diabetes. *Ann. Intern. Med.,* **127,** 788–795.

[2] Alder, V., En, S., Cringle, S., and Yu, P. (1997) Diabetic retinopathy: Early functional changes. *Clin. Exp. Pharmacol. Physiol.,* **24,** 785–788.

[3] Sone, H., Kawakami, Y., Okuda, Y., Sekine, Y., Honmura, S., Matsuo, K., Segawa, T., Suzuki, H., and Yamashita, K. (1997) Ocular vascular endothelial growth factor levels in diabetic rats are elevated before observable retinal proliferative changes. *Diabetologia,* **40,** 726–730.

[4] Qaum, T., Xu, Q., Joussen, A. M., Clemens, M. W., Qin, W., Miyamoto, K., Hassessian, H., Wiegand, S. J., Rudge, J., Yancopoulos, G. D., and Adamis, A. P. (2001) VEGF-initiated blood-retinal barrier breakdown in early diabetes. *Invest. Ophthalmol. Vis. Sci.,* **42,** 2408–2413.

[5] Miyamoto, K., Khosrof, S., Bursell, S. E., Moromizato, Y., Aiello, L. P., Ogura, Y., and Adamis, A. P. (2000) Vascular endothelial growth factor (VEGF)-induced retinal vascular permeability is mediated by intercellular adhesion molecule-1 (ICAM-1). *Am. J. Pathol.,* **156,** 1733–1739.

[6] Antonetti, D. A., Barber, A. J., Khin, S., Lieth, E., Tarbell, J. M., and Gardner, T. W. (1998) Vascular permeability in experimental diabetes is associated with occludin content: Vascular endothelial factor decreases occludin in retinal endothelial cells. *Diabetes,* **47,** 1953–1959.

[7] Sharp, P. (1995) The role of growth factors in the development of diabetic retinopathy. *Metabolism,* **44,** 72–75.

[8] Pfeiffer, A., Spranger, J., Meyer-Schwickerath, R., and Schatz, H. (1997) Growth factor alterations in advanced diabetic retinopathy: A possible role of blood retina barrier breakdown. *Diabetes,* **2**(Suppl), S26–S30.

[9] Simo, R., Lecube, A., Segura, R. M., Garcia-Arumi, J., and Hernandez, C. (2002) Free insulin growth factor-I and vascular endothelial growth factor in the vitreous fluid of patients with proliferative diabetic retinopathy. *Am. J. Ophthalmol.,* **134,** 376–382.

[10] Ismail, G. M., and Whitaker, D. (1998) Early detection of changes in visual function in diabetes mellitus. *Ophthalmic Physiol. Opt.,* **18,** 3–12.

[11] Ishii, D. N. (1995) Implication of insulin-like growth factors in the pathogenesis of diabetic neuropathy. *Brain Res. Rev.,* **20,** 47–67.

[12] Zhuang, H.-X., Snyder, C. K., Pu, S.-F., and Ishii, D. N. (1996) Insulin-like growth factors reverse or arrest diabetic neuropathy: Effects on hyperalgesia and impaired nerve regeneration in rats. *Exp. Neurol.,* **140,** 198–205.

[13] Barber, A. J., Lieth, E., Khin, S. A., Antonetti, D. A., Buchanan, A. G., and Gardner, T. W. (1998) Neural apoptosis in the retina during experimental and human diabetes. Early onset and effect of insulin. *J. Clin. Invest.,* **102,** 783–791.

[14] Zeng, X. X., Ng, Y. K., and Ling, E. A. (2000) Neuronal and microglial response in the retina of streptozotocin-induced diabetic rats. *Vis. Neurosci.,* **17,** 463–471.

[15] Ishii, D. N., and Lupien, S. B. (1995) Insulin-like growth factors protect against diabetic neuropathy: Effects on sensory nerve regeneration in rats. *J. Neurosci. Res.,* **40,** 138–144.

[16] Zhuang, H.-X., Wuarin, L., Fei, Z.-J., and Ishii, D. N. (1997) Insulin-like growth factor (IGF) gene expression is reduced in neural tissues and liver from rats with non-insulin-dependent diabetes mellitus, and IGF treatment ameliorates diabetic neuropathy. *J. Pharmacol. Exp. Ther.,* **283,** 366–374.

[17] Schmidt, R. E., Dorsey, D. A., Beaudet, L. N., Plurad, S. B., Parvin, C. A., and Miller, M. S. (1999) Insulin-like growth factor I reverses experimental diabetic autonomic neuropathy. *Am. J. Pathol.,* **155,** 1651–1660.

[18] Seigel, G. M., Chiu, L., and Paxhia, A. (2000) Inhibition of neuroretinal cell death by insulin-like growth factor-1 and its analogs. *Mol. Vis.,* **6,** 157–163.

[19] Politi, L. E., Rotstein, N. P., Salvador, G., Giusto, N. M., and Insua, M. F. (2001) Insulin-like growth factor-I is a potential trophic factor for amacrine cells. *J. Neurochem.,* **76,** 1199–1211.

[20] Acerini, C. L., Patton, C. M., Savage, M. O., Kernell, A., Westphal, O., and Dunger, D. B. (1997) Randomised placebo-controlled trial of human recombinant insulin-like growth factor I plus intensive insulin therapy in adolescents with insulin-dependent diabetes mellitus. *Lancet,* **350,** 1199–1204.

[21] Castellon, R., Hamdi, H. K., Sacerio, I., Aoki, A. M., Kenney, M. C., and Ljubimov, A. V. (2002) Effects of angiogenic growth factor combinations on retinal endothelial cells. *Exp. Eye Res.,* **74,** 523–535.

[22] Near, S. L., Whalen, L. R., Miller, J. A., and Ishii, D. N. (1992) Insulin-like growth factor II stimulates motor nerve regeneration in rats. *Proc. Natl. Acad. Sci. U.S.A.,* **89,** 11716–11720.

[23] Glazner, G. W., Lupien, S., Miller, J. A., and Ishii, D. N. (1993) Insulin-like growth factor-II increases the rate of sciatic nerve regeneration in rats. *Neuroscience,* **54,** 791–797.

[24] Pu, S.-F., Zhuang, H.-X., Marsh, D. J., and Ishii, D. N. (1999) Insulin-like growth factor-II increases and IGF is required for postnatal rat spinal motoneuron survival following sciatic nerve axotomy. *J. Neurosci. Res.,* **55,** 9–16.

[25] Gao, W.-Q., Shinsky, N., Ingle, G., Beck, K., Elias, K. A., and Powell-Braxton, L. (1999) IGF-I deficient mice show reduced peripheral nerve conduction velocities and decreased axonal diameters and respond to exogenous IGF-I treatment. *J. Neurobiol.,* **39,** 142–152.

[26] Beck, K., Powell-Braxton, L., Widmer, H., Valverde, J., and Hefti, F. (1995) IGF1 gene disruption results in reduced brain size, CNS hypomyelination, and loss of hippocampal granule and striatal parvalbumin-containing neurons. *Neuron,* **14,** 717–730.

[27] Ishii, D. N., Guertin, D. M., and Whalen, L. R. (1994) Reduced insulin-like growth factor I mRNA content in liver, adrenal glands and spinal cord of diabetic rats. *Diabetologia,* **37,** 1073–1081.

[28] Wuarin, L., Guertin, D. M., and Ishii, D. N. (1994) Reduction in insulin-like growth factor (IGF) gene expression in nerves precedes the onset of diabetic neuropathy. *Exp. Neurol.* **130,** 106–114.

[29] Lowe, W. L. Jr., Florkiewicz, R. Z., Yorek, M. A., Spanheimer, R. G., and Albrecht, B. N. (1995) Regulation of growth factor mRNA levels in the eyes of diabetic rats. *Metabolism,* **44,** 1038.

[30] Bornfeldt, K. E., Arnqvist, H. J., Enberg, B., Mathews, L. S., and Norstedt, G. (1989) Regulation of insulin-like growth factor-I and growth hormone receptor gene expression by diabetes and nutritional state in rat tissues. *J. Endocrinol.,* **122,** 651–656.

[31] Fagin, J. A., Roberts, C. T. Jr., LeRoith, D., and Brown, A. T. (1989) Coordinate decrease of tissue insulin-like growth factor I posttranscriptional alternative mRNA transcripts in diabetes mellitus. *Diabetes,* **38,** 428–434.

[32] Gilbert, R. E., Vranes, D., Berka, J. L., Kelly, D. J., Cox, A., Wu, L. L., Stacker, S. A., and Cooper, M. E. (1998) Vascular endothelial growth factor and its receptors in control and diabetic rat eyes. *Lab. Invest.,* **78,** 1017–1027.

[33] Gillespie, C., Read, L. C., Bagley, C. J., and Ballard, F. J. (1990) Enhanced potency of truncated insulin-like growth factor-I (des(1–3)IGF-I) relative to IGF-I in lit/lit mice. *J. Endocrinol.,* **127,** 401–405.

[34] Tan, K., and Baxter, R. C. (1986) Serum insulin-like growth factor I levels in adult diabetic patients: The effect of age. *J. Clin. Endocrinol. Metab.,* **63,** 651–655.

[35] Arner, P., Sjoberg, S., Gjotterberg, M., and Skottner, A. (1989) Circulating insulin-like growth factor I in type 1 (insulin-dependent) diabetic patients with retinopathy. *Diabetologia,* **32,** 753–758.

[36] Sara, V., Carlsson-Skwirut, C., Anderson, C., Hall, E., Sjogren, B., Holmgren, A., and Jornvall, H. (1986) Characterization of somatomedins from human fetal brain: identification of a variant form of insulin-like growth factor I. *Proc. Natl. Acad. Sci. U.S.A.,* **83,** 4904–4907.

[37] Carlsson-Skwirut, C., Jornvall, H., Holmgren, A., Andersson, C., Bergman, T., Lundquist, G., Sjogren, B., and Sara, V. (1986) Isolation and characterization of variant IGF-I as well as IGF-2 from adult human brain. *FEBS Lett.,* **201,** 46–50.

[38] Carlsson-Skwirut, C., Lake, M., Hartmanis, M., Hall, K., and Sara, V. R. (1989) A comparison of the biological activity of the recombinant intact and truncated insulin-like growth factor I. *Biochim. Biophys. Acta.,* **1011,** 192–197.

[39] Heding, A., Gill, R., Ogawa, Y., DeMeyts, P., and Shymko, R. M. (1996) Biosensor measurement of the binding of insulin-like growth factors I and II and their analogues to the insulin-like growth factor-binding protein-3. *J. Biol. Chem.,* **271,** 13948–13952.

[40] Bagley, C. J., May, B., Szabo, L., McNamara, P. J., Ross, M., Francis, G. L., Ballard, F. J., and Wallace, J. C. (1989) A key functional role for the insulin-like growth factor 1 N-terminal pentapeptide. *Biochem. J.,* **259,** 665–671.

[41] Clemmons, D. R., Camacho-Hubner, C., McCusker, R. H., and Bayne, M. L. (1990) Discrete alterations of the insulin-like growth factor I molecule which alters its affinity for insulin-like growth factor binding proteins in changes in bioactivity. *J. Biol. Chem.,* **265,** 12210–12216.

[42] Ballard, F. J., Francis, G. L., Ross, M., Bagley, C. J., May, B., and Wallace, J. C. (1987) Natural and synthetic forms of insulin-like growth factor-1 (IGF-1) and the potent derivative, destripeptide IGF-1: Biological activities and receptor binding. *Biochem. Biophys. Res. Commun.,* **149,** 398–404.

[43] Gerhardinger, C., McClure, K. D., Romero, G., Podesta, F., and Lorenzi, M. (2001) IGF-I mRNA and signaling in the diabetic retina. *Diabetes,* **50,** 175–183.

[44] Burgos, R., Mateo, C., Canton, A., Hernandez, C., Mesa, J., and Simo, R. (2000). Vitreous levels of IGF-I, IGF binding protein 1, and IGF binding protein 3 in proliferative diabetic retinopathy. *Diabetes Care,* **23,** 80–83.

[45] Spranger, J., Buhnen, J., Jansen, V., Krieg, M., Meyer-Schwickerath, R., Blum, W. F., Schatz, H., and Pfeiffer, A. F. (2000) Systemic levels contribute significantly to increased intraocular IGF-I, IGF-II and IGF-BP3 in proliferative diabetic retinopathy. *Horm. Metab. Res.,* **32,** 196–200.

[46] Bitar, M. S., Pilcher, C. W. T., Khan, I., and Waldbillig, R. J. (1997) Diabetes-induced suppression of IGF-I and its receptor mRNA levels in rat superior cervical ganglia (1997). *Diabetes Res. Clin. Pract.,* **38,** 73–80.

[47] Werner, H., Shen-Orr, Z., Stannard, B., Burguera, B., Roberts, C. T. Jr., and LeRoith, D. (1990) Experimental diabetes increases insulinlike growth actor I and II receptor concentration and gene expression in kidney. *Diabetes,* **39,** 1490–1497.

[48] Bornfeldt, K. E., Skottner, A., and Arnqvist, H. J. (1992) In-vivo regulation of messenger RNA encoding insulin-like growth factor-I (IGF-I) and its receptor by diabetes, insulin and IGF-I in rat muscle. *J. Endocrinol.,* **135,** 203–211.

[49] Li, Z.-G., Zhang, W., Grunberger, G., and Sima, A. A. F. (2002) Hippocampal neuronal apoptosis in type 1 diabetes. *Brain Res.,* **946,** 221–231.

[50] Spoerri, P. E., Ellis, E. A., Tarnuzzer, R. W., and Grant, M. B. (1998) Insulin-like growth factor: receptor and binding proteins in human retinal endothelial cell cultures of diabetic and non-diabetic origin. *Growth Horm. IGF Res.,* **8,** 125–132.

[51] Ozaki, H., Seo, M. S., Ozaki, K., Yamada, H., Yamada, E., Okamoto, N., Hofmann, F., Wood, J. M., and Campochiaro, P. A. (2000) Blockade of vascular endothelial cell growth factor receptor signaling is sufficient to completely prevent retinal neovascularization. *Am. J. Pathol.,* **156,** 697–707.

[52] Shinoda, K., Ishida, S., Kawashima, S., Wakabayashi, T., Uchita, M., Matsuzaki, T., Takayama, M., Shinmura, K., and Yamada, M. (2000) Clinical factors related to the aqueous levels of vascular endothelial growth factor and hepatocyte growth factor in proliferative diabetic retinopathy. *Curr. Eye Res.,* **21,** 655–661.

[53] Pe'er, J., Folberg, R., Itin, A., Gnessin, H., Hemo, I., and Keshet, E. (1996) Upregulated expression of vascular endothelial growth factor in proliferative diabetic retinopathy. *Br. J. Ophthalmol.,* **80,** 241–245.

[54] Amin, R. H., Frank, R. N., Kennedy, A., Eliott, D., Puklin, J. E., and Abrams, G. W. (1997) Vascular endothelial growth factor is present in glial cells of the retina and optic nerve of human subjects with nonproliferative retinopathy. *Invest. Ophthalmol. Vis. Sci.,* **38,** 36–47.

[55] Hammes, H. P., Lin, J., Bretzel, R. G., Brownlee, M., and Breier, G. (1998) Upregulation of the vascular endothelial growth factor/vascular endothelial growth factor receptor system in experimental background diabetic retinopathy of the rat. *Diabetes,* **47,** 401–406.

[56] Murata, T., Nakagawa, K., Khalil, A., Ishibashi, T., Inomata, H., and Sueishi, K. (1996) The relation between expression of vascular endothelial growth factor and breakdown of the blood-retinal barrier in diabetic rat retinas. *Lab. Invest.,* **74,** 819–825.

[57] Obrosova, I. G., Minchenenko, A. G., Marinescu, V., Fathallah, L., Kennedy, A., Stockert, C. M., Frank, R. N., and Stevens, M. J. (2001) Anti-oxidants attenuate early up regulation of retinal vascular endothelial growth factor in streptozotocin rats. *Diabetologia,* **44,** 1102–1110.

[58] Urata, Y., Yamagucho, M., Higashiyama, Y., Ihara, Y., Goto, S., Kuwano, M., Horiuchi, S., Sumikawa, K., and Kondo, T. (2002) Reactive oxygen species accelerate production of vascular endothelial growth factor by advanced glycation end products in RAW264.7 mouse macrophages. *Free Radic. Biol. Med.,* **32,** 688–701.

[59] Tilton, R. G. (2002) Diabetic vascular dysfunction: Links to glucose-induced reductive stress and VEGF. *Microsc Res. Tech.,* **57,** 390–407.

[60] Seigel, G. M., Lupien, S., Campbell, L. M., and Ishii, D. N. (2003) Systemic IGF-1 treatment inhibits neuroretinal cell death in diabetic rat retina. *Assoc. Res. Vis. Ophthamol.,* in press.

[61] Plas, D. R., and Thompson, C. B. (2002) Cell metabolism and the regulation of programmed cell death. *Trends Endocrinol. Metab.,* **13,** 74–78.

[62] Lawlor, M. A., and Alessi, D. (2001) PKB/Akt: A key mediator of cell proliferation, survival and insulin responses? *J. Cell Sci.,* **114,** 2903–2910.

[63] Walker, K. S., Deak, M., Paterson, A., Hudson, K., Cohen, P., and Alessi, D. R. (1998) Activation of protein kinase B beta and gamma isoforms by insulin in vivo and by 3-phosphoinositide-dependent protein kinase in vitro: comparison with protein kinase B alpha. *Biochem. J.,* **331,** 299–308.

[64] Cho, H., Mu, H., Kim, J. K., Thorvaldsen, J. L., Chu, Q., Crenshaw, E. B., Kaestner, K. H., Bartolomei, M. S., Shulman, G. I., and Burnbaum, M. J. (2001) Insulin resistance and diabetes mellitus-like syndrome in mice lacking protein kinase Akt2 (PKB-beta). *Science,* **292,** 1728–1731.

[65] Suzuma, I., Kiyoshi, S., Ueki, K., Hata, Y., Feener, E. P., King, G. L., and Aiello, L. P. (2002) Stretch-induced retinal vascular endothelial growth factor expression is mediated by phosphatidyl inositol 3-kinase and protein kinase C (PKC)-$\zeta$ but not by stretch-induced ERK1/2, Akt, Ras, or classic/novel PKC pathways. *J. Biol. Chem.,* **277,** 1047–1057.

[66] Downward, J. (1998). Mechanisms and consequences of activation of protein kinase B/Akt. *Curr. Opin. Cell Biol.,* **10,** 262–267.

[67] Pulford, B. E., and Ishii, D. N. (2001) Uptake of circulating insulin-like growth factors (IGFs) into cerebrospinal fluid appears to be independent of the IGF receptors as well as IGF-binding proteins. *Endocrinology,* **142,** 213–220.

[68] Barber, A., Nakamura, M., Wolpert, E. B., Reiter, C., Seigel, G. M., Antonetti, D. A., and Gardner, T. A. (2001) Insulin rescues retinal neurons from apoptosis by a phosphotidylinositol 3-kinase/Akt-mediated mechanism that reduces the activation of caspase-3. *J. Biol. Chem.,* **276,** 32814–32821.

